# The Mitochondrial Unfolded Protein Response Protects against Anoxia in *Caenorhabditis elegans*

**DOI:** 10.1371/journal.pone.0159989

**Published:** 2016-07-26

**Authors:** Salvador Peña, Teresa Sherman, Paul S. Brookes, Keith Nehrke

**Affiliations:** 1 Department of Pathology and Laboratory Medicine, University of Rochester Medical Center, Rochester, New York, United States of America; 2 Department of Medicine, University of Rochester Medical Center, Rochester, New York, United States of America; 3 Department of Anesthesiology, University of Rochester Medical Center, Rochester, New York, United States of America; 4 Department of Pharmacology and Physiology, University of Rochester Medical Center, Rochester, New York, United States of America; Albany Medical College, UNITED STATES

## Abstract

The mitochondrial unfolded protein response (UPR^mt^) is a surveillance pathway that defends proteostasis in the “powerhouse” of the cell. Activation of the UPR^mt^ protects against stresses imposed by reactive oxygen species, respiratory chain deficits, and pathologic bacteria. Consistent with the UPR^mt^’s role in adaption, we found that either its pharmacological or genetic activation by ethidium bromide (EtBr) or RNAi of the mitochondrial AAA-protease *spg-7* was sufficient to reduce death in an anoxia-based *Caenorhabditis elegans* model of ischemia-reperfusion injury. The UPR^mt^-specific transcription factor *atfs-1* was necessary for protection and *atfs-1* gain-of-function (gf) mutants were endogenously protected from both death and dysfunction. Neurons exhibited less axonal degeneration following non-lethal anoxia-reperfusion (A-R) when the UPR^mt^ was pre-activated, and consistent with the concept of mitochondrial stress leading to cell non-autonomous (ie. “remote”) effects, we found that restricted activation of the UPR^mt^ in neurons decreased A-R death. However, expression of the *atfs-1(gf)* mutant in neurons, which resulted in a robust activation of a neuronal UPR^mt^, did not upregulate the UPR^mt^ in distal tissues, nor did it protect the worms from A-R toxicity. These findings suggest that remote signaling requires additional component(s) acting downstream of *de facto* mitochondrial stress.

## Introduction

The mitochondrial unfolded protein response (UPR^mt^) is an adaptive signaling pathway that was first identified in mammals [1], but has been best characterized genetically in the nematode *Caenorhabditis elegans* [2–5]. Misfolded proteins in the mitochondria trigger the expression of a repertoire of genes that relieve respiratory burden and restore proteostasis (for review, see reference [6]). Activation of the UPR^mt^ occurs in response to disruptions in the stoichiometric equilibrium between nuclear and mitochondria-encoded electron transport chain (ETC) subunits [7], reactive oxygen species (ROS) [8, 9], and by exposure to pathogenic bacteria [10], suggesting that it is part of a surveillance mechanism that responds to disruption of core physiologic processes [8, 10–13]. Interestingly, mitochondrial stress in *C*. *elegans* neurons has been shown to activate the UPR^mt^ in distal tissues and to regulate lifespan, leading to the idea that mitochondrial proteostasis is a sounding board that can trigger adaptation remotely through cell-cell communication [14].

In *C*. *elegans*, mito-nuclear retrograde signaling via the bZip transcription factor, ATFS-1, is a critical component of the UPR^mt^ axis [15, 16]. ATFS-1 normally traffics to mitochondria, however during mitochondrial dysfunction a fraction of ATFS-1 fails to reach the mitochondria and instead accumulates in the nucleus. Additionally, the UPR^mt^ is regulated by dimerization and nuclear localization of the *dve-1* encoded transcription factor along with a small ubiquitin like protein *ubl-5*, as well as the activity of mTOR regulator, *rheb-1*. [2, 3]. Importantly, ATFS-1 coordinates mito-nuclear genomic output to properly balance the stoichiometry of electron transport chain complex assembly in order to maintain oxidative phosphorylation during mitochondrial stress [15]. Several mitochondrial electron transport chain (ETC) mutants show UPR^mt^ activation and require *atfs-1* for survival [16] suggesting that this signaling pathway helps the organism to cope with mitochondrial dysfunction.

In the cellular stress imposed by oxygen deprivation, mitochondria are central to both death and survival (for review, see reference [17]). Significant events leading to death are irreversible inhibition of oxidative phosphorylation, proton leak across the mitochondrial inner membrane, calcium overload, reactive oxygen species (ROS) generation and permeability transition pore opening [18–23]. However, interventions that preserve mitochondria function, for example by scavenging ROS or inhibiting PT pore opening, are protective [24–26].

Recently, anoxia-reperfusion (A-R) has been shown to cause the accumulation of misfolded proteins in the mitochondria of *C*. *elegans* [27]. A-R has been used extensively as a surrogate for ischemia-reperfusion in worms, due to their diminished sensitivity to low oxygen levels compared to mammals [28]. Protecting the mitochondrial protein folding environment by simply over expressing mitochondrial molecular chaperones (Hsp-60 and mtHsp-70) can protect mammalian cells from ischemia-reperfusion (I-R) injury [29, 30] and inhibiting mitochondrial translation through knockdown of aminoacyl-tRNAs has been shown to protect against A-R injury in worms [31]. Collectively, it seems that interventions that support mitochondrial proteostasis can protect against the detrimental effects of low oxygen. In fact, pre-activation of the UPR^mt^ was recently shown to protect against anoxia in worms [27].

Herein, we build on the foundation that *atfs-1* is required for protection from A-R injury, to show that *atfs-1* gain-of-function (gf) mutants that exhibit constitutive activation of the UPR^mt^ are endogenously protected. Based on this result, we hypothesized that protection might occur through intrinsic, cell autonomous processes or through remote effects that are coordinated throughout the organism (ie, “mitokine” signaling). To address this idea, we developed of a novel genetic model for single-copy cell restricted expression of an *atfs-1(gf)* mutant. Our results indicate that cell autonomous protection from A-R injury can be elicited through an *atfs-1(gf)*, but that remote UPR^mt^ activation and protection from death may require other pathway(s) that responds to *de facto* mitochondrial stress.

## Materials and Methods

### Strains

Worm strains were routinely propagated using standard culture techniques at 20°C on nematode growth media (NGM) agar plates containing 5 μg/ml cholesterol and seeded with OP50 bacteria, as described [32]. The strains used in this work are:

SJ4058, zcIs9 [*Phsp-60*::*GFP*] V; CMH5, *atfs-1(tm4525)* V; QC115, *atfs-1(et15)* V; EG4322, ttTi5605 II; *unc-119(ed9)* III;; KWN176, rnyls13 [*Pmec-4*::*mCherry; unc-119(+)*] X; KWN 456, rnySi37 [*Patfs-1*::*wCherry*::*atfs-1*::*GFP*, *cb-unc-119(+)*] II; KWN 495, *pha-1 (e2123ts)* III, *him-5 (e1490)* V, rnyEx289 [*Patfs-1*::*flp*, *Pmyo-2*::*mCherry*, *pha-1(+)*]; KWN484, *atfs-1(tm4525)* V, rnySi37, zcIs9, rnyEx289; KWN525 rnySi49 [*Patfs-1*::*wCherry*::*atfs-1(et15)*::*GFP*, *cb-unc-119(+)*] II; *unc-119(ed3)* III; KWN529, *pha-1 (e2123ts)* III, *him-5 (e1490)* V, rnyEx297 [*Prab-3*::*flp*, *Pmyo-2*::*mCherry*, *pha-1(+)*]; KWN566, rnyls13, zcIs9; KWN567, *atfs-1(tm4525)*, rnyls13, zcIs9; KWN568, *atfs-1(et15)*, rnyls13, zcIs9; KWN562, *atfs-1(tm4525)*, rnySi49, zcIs9, rnyEx297; KWN569, rnySi49, zcIs9, rnyEx297; KWN591, *atfs-1(tm4525)*, rnySi49, zcIs9, rnyls013, rnyEx289; KWN590, rnySi49, zcIs9, rnyls013, rnyEx289; HC196, *sid-1(qt9)*; AGD724, *sid-1(qt9)*, uthIs243 [*Prab-3*::*cco-1HP*, *Pmyo-2*::*tdTomato*]

Standard genetic protocols were used for crosses. The zcIs9 insertion on chromosome V is closely linked to *atfs-1*. Hence, GFP signal was used to follow *atfs-1* following mating. To confirm that the genomic *atfs-1* allele co-segregated with zcIs9, the final strains were PCR genotyped using primers 5’-GCCTCCTTTCGCCTTTTGTCATC-3’, 5’- GCACAGCTTCTCCGATTCAGTG-3’, and 5’-gggggatttttagtcggcaatg-3’. These primers do not amplify the chromosome II MosSCI insertions. PCR products were sequenced to identify the *atfs-1(et15)* allele, which is a missense mutation. MosSCI insertions were confirmed using primers OG967, 5’-AGGCAGAATGTGAACAAGACTCG-3’ and OG970, 5’-ATCGGGAGGCGAACCTAACTG-3’, as described [33].

### Molecular Biology

The *atfs-1* promoter (~2.5 kb) was PCR amplified from genomic DNA using primers 5’-ACACGTCGACCAAACAATTTGATGGTACTGTTTCAGAT-3' and 5'-ACACCCTAGGCGAAGTTACACCTGCAAATGTACAAG-3' and cloned into pFH6.II-linker [34] as an *Avr*II-*Sal*I fragment to create pSEP1. The 3’ untranslated region from *atfs-1* was PCR amplified from genomic DNA using primers 5’-ACACGCGGCCGCCAATAATCAGAATTCGAAACAATTGTTC-3’ and 5’-ACACACTAGTCCTAGGTTGGCTAAACAGGTAACG-3’ and inserted into pSEP1 as a *Not*I-*Spe*I fragment to create pSEP2. A *Sal*I-*Acc65*I FRT-wCherry-FRT fragment was cloned from pWD176-myo-2-flop-wCherry [9] into pSEP2 to create pSEP3. The *atfs-1* open reading frame was PCR amplified from wild-type genomic DNA with primers 5’-ACACGGTACCAAAAATGTTTTCCCGTGTGGGACGTCTC-3’ and 5’-ACACGGTACCTGAATAATGGCGCCCATTTTACGAAG-3’ and inserted into pSEP3 as an *Acc65*I fragment to create pSEP4. The *atfs-1(et15)* open reading frame was PCR amplified using identical primers and inserted into pSEP3 to create pSEP6. pSEP4 and pSEP6 were digested with *Avr*II-*Spe*I and cloned into the *Spe*I site in the MosSCI vector pCFJ151-p5605 [33] to create pSEP5 and pSEP7, respectively. The *Age*I-*Avr*II fragment from pWD79-hsp-2u-flp-RV (obtained courtesy of Dr. Wayne Davies) was cloned into the *Age*I-*Spe*I sites of pFH6.II in place of GFP to create pFLF1. The *rab-3* promoter was PCR amplified from genomic DNA using primers 5’-ATATGTCGACCAGCCGCAATCTGAAAATAGGGCTACTGTAG-3’ and 5’-ATATCCTAGGGACGACGACGACCTCGACGG-3’ and cloned into pFLF1 as a *Sal*I-*Avr*lI fragment to create pSEP10. The *atfs-1* promoter was also cloned into pFLF1 to create pSEP11. All PCR-amplified fragments were sequenced to verify fidelity.

### Transgenesis and MosSCI Single Copy Gene Insertion

Injection of DNA into the gonads of young adult animals was performed as described [35]. The protocol for single copy insertion has been similarly well described [33, 36]. Briefly, a strain containing the Mos transposon ttTi6505 in chromosome II was injected with pSEP5 or pSEP7, along with vectors coding for positive and negative selectable markers and an inducible Mos transposase. Following recombination between pSEP5 or pSEP7 and the ttTi5605 site, the chromosome II integration locus was PCR amplified and sequenced to confirm the presence of a full-length insert. All resulting strains were backcrossed to wildtype N2 prior to use.

### Anoxia-Reperfusion

Developmentally-synchronized populations of hermaphrodites were established by using alkaline-bleach treatment (20% commercial bleach, 500 mM NaOH) to generate embryos from gravid adults. These embryos were then cultured under standard conditions at 20°C until adulthood, which was defined as 1 day after vulva development was complete, then subject to anoxia. For experiments examining axonal degeneration, staged L1/L2 larvae (24 hours post-hatch) were used in place of adults. Experimental anoxia was established as described [37]. Briefly, the worms were washed 3x in M9 solution (22 mM KH_2_PO_4_, 42 mM Na_2_HPO_4_, 86 mM NaCl, 1mM MgSO_4_, pH 7) and transferred to an anaerobic chamber (Coy Lab Products, USA) equipped with a palladium catalyst to scavenge oxygen (O_2_<0.01 ppm) for 20 h under 95% N_2_ and 5% H_2_ at ~26°C. Worms were scored for viability 20–24 hours after anoxic exposure as previously described [38]. In general, 2–6 replicates (or individual plates) were assayed per day per condition. The percentage lethality from each plate was then averaged to create a single data point, representing from 49–946 worms (average of 223 worms per data point; see S1 Table for raw data). Experimental manipulations were always assessed in parallel with controls, in order to normalize to index lethality, which fluctuated on a daily basis and was the greatest source of experimental variability. For presentation purposes, the values from individual paired trials were plotted side-by-side, together with their averages.

### RNA Interference

Synchronized larval stage 2/3 worms were moved to plates supplemented with transformed HT115 E.coli bacteria where an *spg-7* insert flanked by T7 promoter sites or empty vector control (pPD129.36) was induced using IPTG. Worms were incubated on these plates for at least 24 hours prior to experimental use.

### Imaging

Worms were imaged on a 2% agarose pad under anesthetic (0.1% tetramisole). Neuronal puncta were visualized from transgenic worms expressing a red mCherry fusion in mechanosensory neurons, in the L2–L3 stage as previously described [39], using a Nikon Eclipse TE2000-U microscope (Nikon USA, Melville, NY, USA) with a Polychrome V monochromator (TILL Photonics, Gräfelfing, Germany) running TILLvisION software. Neuronal cell bodies were identified under a Texas Red filter set using a 10x objective, then viewed at higher magnification, typically with a 100x oil objective, to assess axonal breaks and abnormalities. Most of these consisted of puncta where the neuronal process was visibly enlarged and at least 2-fold more fluorescent than the surrounding process. The number of abnormalities in either the PVML or PVMR, one of two mechanosensory neurons in the posterior of the worm, was scored in a region extending from the cell body to the vulva. These processes under normal conditions are extremely homogenous with respect to the distribution of fluorescent reporter. Since they run close to the surface or “skin” of the worm, they are easily scored. In general, ten worms were analyzed per day, the data was averaged, and the experiments were repeated 3–4 times (see S1 Table for raw data). Other strains expressing various gene fusions, reporters, or fluorescent markers were examined using an Olympus FV1000 confocal microscope (available as part of the University of Rochester Confocal Core) under appropriate illumination.

### Statistics

Statistical significance was determined through Student’s *t* tests, comparing paired trials. “N” refers to the number of trials (and hence paired experimental replicates) analyzed, each consisting of > twenty worms.

## Results

Mitochondria are pivotal for determining survival following exposure to low oxygen levels, and ROS generated under these conditions can lead to both damage and adaptation. Worms are resistant to hypoxia [28] and hence A-R has been utilized to simulate I-R in this genetic model organism [38, 40]. In addition, worms are normally cultivated from 15–25°C and the A-R model utilizes a modestly elevated temperature (26°C) to sensitize the worms to anoxic toxicity. Hence, it is possible that interventions that modulate A-R toxicity may have their basis in thermal tolerance. However, this is true in mammalian systems, as well, where I-R injury is also temperature sensitive, and there is a large body of evidence suggesting that A-R translates well as a surrogate for I-R in mammalian cells (for review, see reference [41]). Here, we utilized *C*. *elegans* to determine the influence of UPR^mt^ activation on surviving A-R.

**Fig 1:**
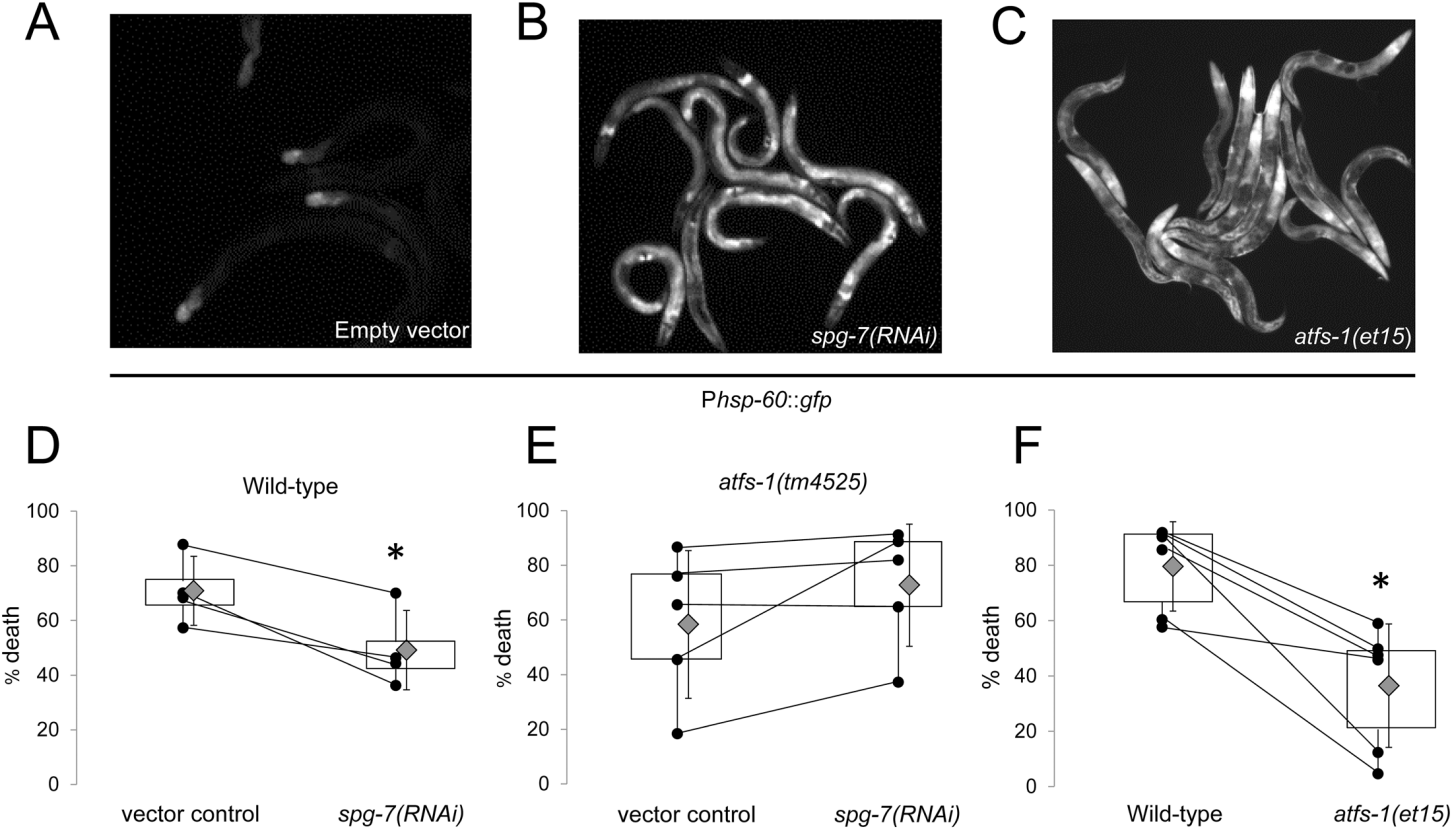
*Atfs-1* is both necessary and sufficient for UPR^mt^ activation and reduced death from anoxia-reperfusion. Representative fluorescent photomicrographs of P*hsp60*::*GFP* activation in A.) vector control and B.) UPR^mt^ inducer *spg-7(RNAi)* treated worms and in C.) an *atfs-1(et15)* gain-of-function mutant. D.) Box-and-whisker plot showing that pre-induction of the UPR^mt^ with *spg-7(RNAi)* reduces death following A-R (n = 4, p*[Student’s t test] <0.05). E.) Box-and-whisker plot showing that *spg-7(RNAi)* does not protect *atfs-1(tm4525)* loss-of-function worms from A-R toxicity (n = 5, p*[Student’s t test] <0.05). F.) Box-and-whisker plot showing that *atfs-1(et15)* mutants are constitutively less susceptible to A-R (n = 6, p*[Student’s t test] <0.05). Experimental replicates are shown as black dots; lines between replicates indicate that they were run on the same day. Grey diamonds are means with the error shown as standard deviations.

The UPR^mt^ reporter P*hsp-60*::*GFP* is strongly activated by RNAi mediated knockdown of *spg-7* (*spg-7(RNAi)*), a mitochondrial quality control m-AAA protease (Fig 1A). *A priori* treatment with *spg-7(*RNAi) reduced death following A-R (vector control 71 ± 7%; *spg-7(RNAi)* 49 ± 8%, error is SEM, p = 0.02; Fig 1D), as did 30 mcg/mL EtBr, an established chemical activator of the UPR^mt^ (S1 Fig). Protection elicited from both regiments was lost in the *atfs-1(tm4525)* loss-of-function (lf) mutant (*atfs-1(tm4525)* 58 ± 14%; *spg-7(RNAi)* 73 ± 11%; error is SEM, p = 0.14; Fig 1E).

Next we examined whether activation of *atfs-1* is sufficient for protection. We took advantage of forward genetic screen for mutations that protected against the toxic non-cholesterol effects of statins [42]. The *atfs-1(et15)* gf allele contains a missense mutation in the first six amino acids that is predicted to reduce ATFS-1 mitochondrial import, and the resulting activation of the UPR^mt^ was shown to protect against statin toxicity [42]. As expected, *atfs-1(et15)* mutants exhibited constitutive induction of the UPR^mt^ reporter gene *hsp-60* (Fig 1C). The *atfs-1(et15)* mutants also exhibited decreased death following A-R (wildtype 80 ± 7%; *atfs-1(et15)* 36 ± 10%, error is SEM, p = 0.006; Fig 1F).

**Fig 2:**
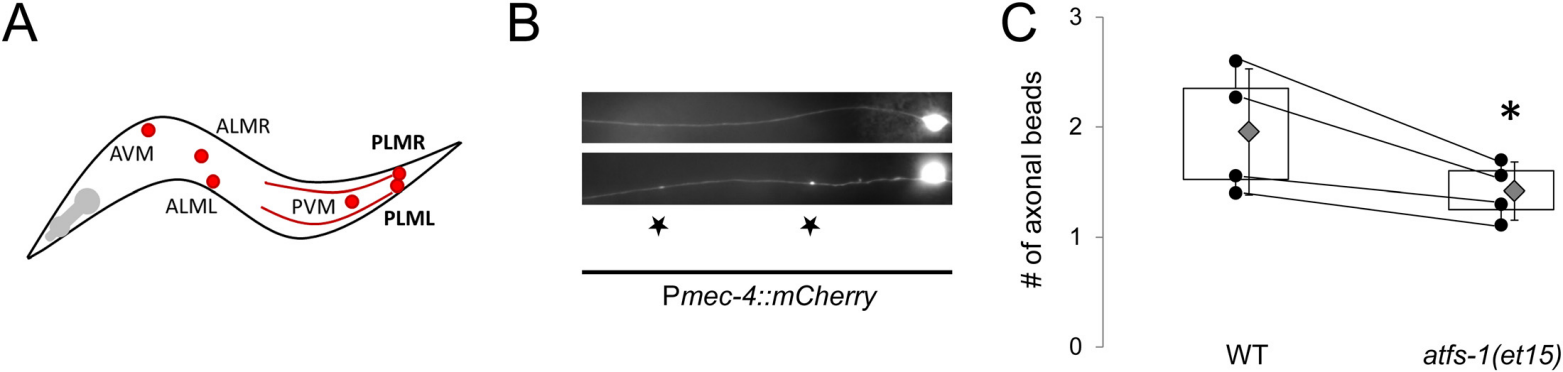
A gain-of-function in *atfs-1* attenuates axonal degeneration following anoxia-reperfusion. A.) Schematic representation of mechanosensory neurons (red dots indicate soma) in the worm, and the PLM axonal processes that were used to measure degeneration. B.) Representative fluorescent photomicrograph of an integrated *Pmec-4*::*mCherry* reporter in wildtype worms (top image) and following A-R (bottom image). Scored puncta are denoted by stars. C.) *atfs-1(et15)* worms develop fewer puncta following A-R (n = 4 independent trials with ~10 worms per genotype per trial), p*[Student’s t test] <0.05). The error is the standard deviation.

We then determined whether induction of the UPR^mt^ could protect tissues from sub-lethal A-R injury. The fluorescent reporter mCherry was expressed in mechanosensory neurons under control of the *mec-4* promoter in order to measure axonal damage [43] (Fig 2A). Process abnormalities including breaks and “bead-on-a-string” type puncta (Fig 2B) have been reported to occur following A-R in these neurons [38]. Following A-R in young larva, which are less susceptible to A-R than adults and hence were used here as a sub-lethal model, *atfs-1(gf)* mutants formed fewer axonal puncta than control animals (wildtype 2.4 ± 0.6; *atfs-1(et15)* 1.4 ± 0.3, error is SD, p = 0.02; Fig 2C). While the difference in puncta between genotypes was small, which is expected given the nature of the model, the data is robust and the difference statistically significant. Previous reports have suggested that upwards of 10 puncta may form following A-R [38]; however, in our hand the only worms that exhibited this many abnormalities were dead or close to death and hence unsuitable for analysis. It is also possible that the differences we observe are due to labeling with mCherry rather than GFP, as was used previously [38].

**Fig 3:**
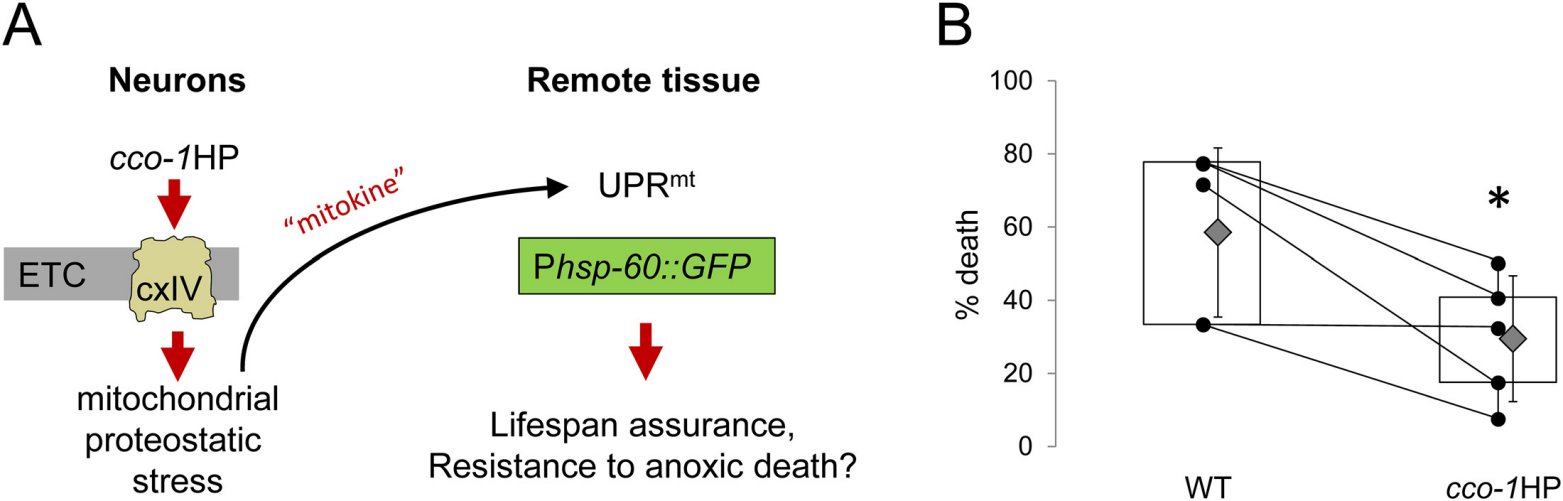
Electron transport chain dysfunction in neurons reduces anoxia-reperfusion toxicity. A.) Schematic of how *cco-1*HP interference with Complex IV leads to mitochondrial stress in neurons, which is transduced remotely through an unidentified “mitokine” to elicit adaptive signaling measures in distal cells. B.) Box-and-whisker plot showing that neuronal *cco-1*HP reduces death following A-R (n = 5, p*[Student’s t test] <0.05). Experimental replicates are shown as black dots; lines between replicates indicate that they were run on the same day. Grey diamonds are means with the error shown as standard deviations.

Neuronal mitochondrial stress can be sensed remotely, suggesting that cells are able to coordinate adaptive responses based upon communicating the status of their mitochondria (Fig 3A) [14]. We found that a small hairpin loop RNA that targets complex IV (*cco-1*HP) expressed under the control of the pan-neuronal *rab-3* promoter decreased A-R death (wildtype 59 ± 12%; *cco-1*HP 30 ± 9%, error is SEM, p = 0.03; Fig 3B). This suggests that neurons are able to coordinate remote adaptation among the whole organism in response to intrinsic mitochondrial stress. However, the *cco-1*HP model presumably combines *de facto* mitochondrial proteotoxicity through ROS generation with compensatory activation of the UPR^mt^. As such, we were motivated to ask whether neuronal expression of the *atfs-1(gf)* mutant, which exhibits compensation in the absence of stress, would also increase survival.

**Fig 4:**
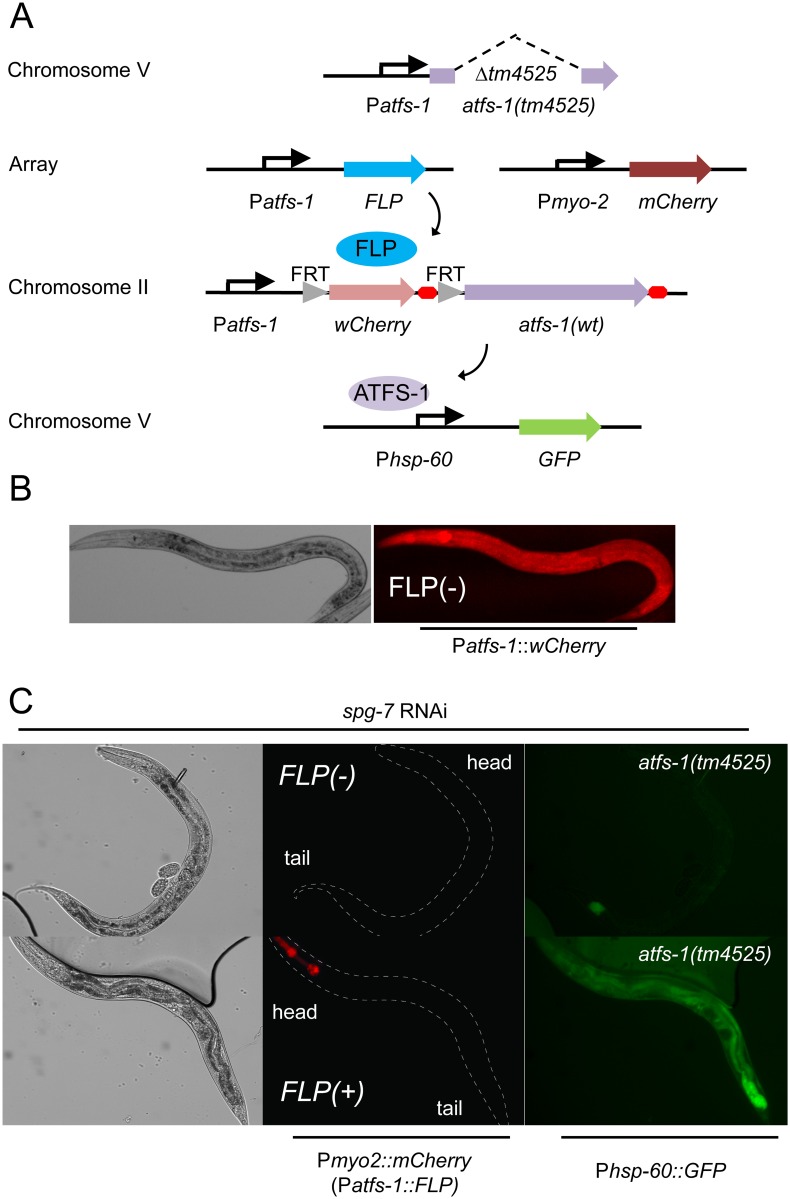
A FLP-out transgenic model to restrict UPR^mt^ activation. A.) Schematic showing relevant genetic constructs to restrict *atfs-1* expression using transgenic FLP recombinase and an *atfs-1* promoter interruption. The activity of the *flp* and *atfs-1* gene products on their target promoters is indicated by arrows. B.) Representative transmission and fluorescent photomicrographs of P*atfs-1*::*wCherry* expressed from a MosSCI single-copy, integrated *atfs-1* promoter interruption in the absence of FLP recombinase. C-E.) Photomicrographs of DIC (grayscale), a fluorescent marker of transgenic FLP (red) and UPR^mt^ reporter P*hsp-60*::*GFP* (green) in *flp*(-) and F-H.) *flp(+)* siblings following *spg-7(RNAi)*. The genomic background is *atfs-1(tm4525)*, a loss-of-function allele.

To achieve this, we generated a novel genetic system that combines two techniques for restricting gene expression. Transgenesis in worms is generally achieved through multi-copy extra-chromosomal arrays that drive recombinant expression. However, the UPR^mt^ reflects a balance between mitochondrial and nuclear ATFS-1 targeting, which is likely to be prone to overexpression artifacts. Moreover, cell-specific promoters exhibit a range of temporal and quantitative differences in expression levels. To circumvent these issues, we first generated a genetic model where FLP recombinase is used to activate gene expression [44]. A recombinant *atfs-1* transgene was cloned where the promoter is interrupted by a *wCherry* insert that is itself flanked by FLP recombinase sites (Fig 4A). This transgene was then integrated via Mos-mediated single copy gene insertion (MosSCI) into the ttTi5605 site on chromosome II [33]. Hence, transgenic ATFS-1 expression should occur at physiological levels from its own promoter but only in a subset of tissues defined by the overlap with a promoter chosen to drive recombinant FLP expression.

Under basal conditions in the absence of FLP recombinase, *wCherry* was widely expressed at low levels (long exposure, Fig 4B), suggesting that the *atfs-1* promoter was active. When the MosSCI *atfs-1(+)* transgene was placed into a genomic *atfs-1(lf)* background, the worms were unable to activate the UPR^mt^ P*hsp-60*::*GFP* reporter in response to *spg-7(RNAi)* (Fig 4C), suggesting effective suppression of ATFS-1 expression by the *wCherry* promoter interruption. In contrast, expression of FLP recombinase from the *atfs-1* promoter itself restored the ability to activate the UPR^mt^ P*hsp-60*::*GFP* reporter (Fig 4C), suggesting effective de-suppression through *wCherry* excision.

In an attempt to discern regulated trafficking of the ATFS-1 transcription factor, a translational GFP fusion was expressed in the MosSCI system, but there was no detectable fluorescent signal even under conditions where we expected to observe increased nuclear trafficking (S3 Fig). We hypothesize that this is likely due to single copy gene expression and the presumed basal degradation of ATFS-1, as its physiologic effects in the nucleus are clearly observed (Fig 4C).

**Fig 5:**
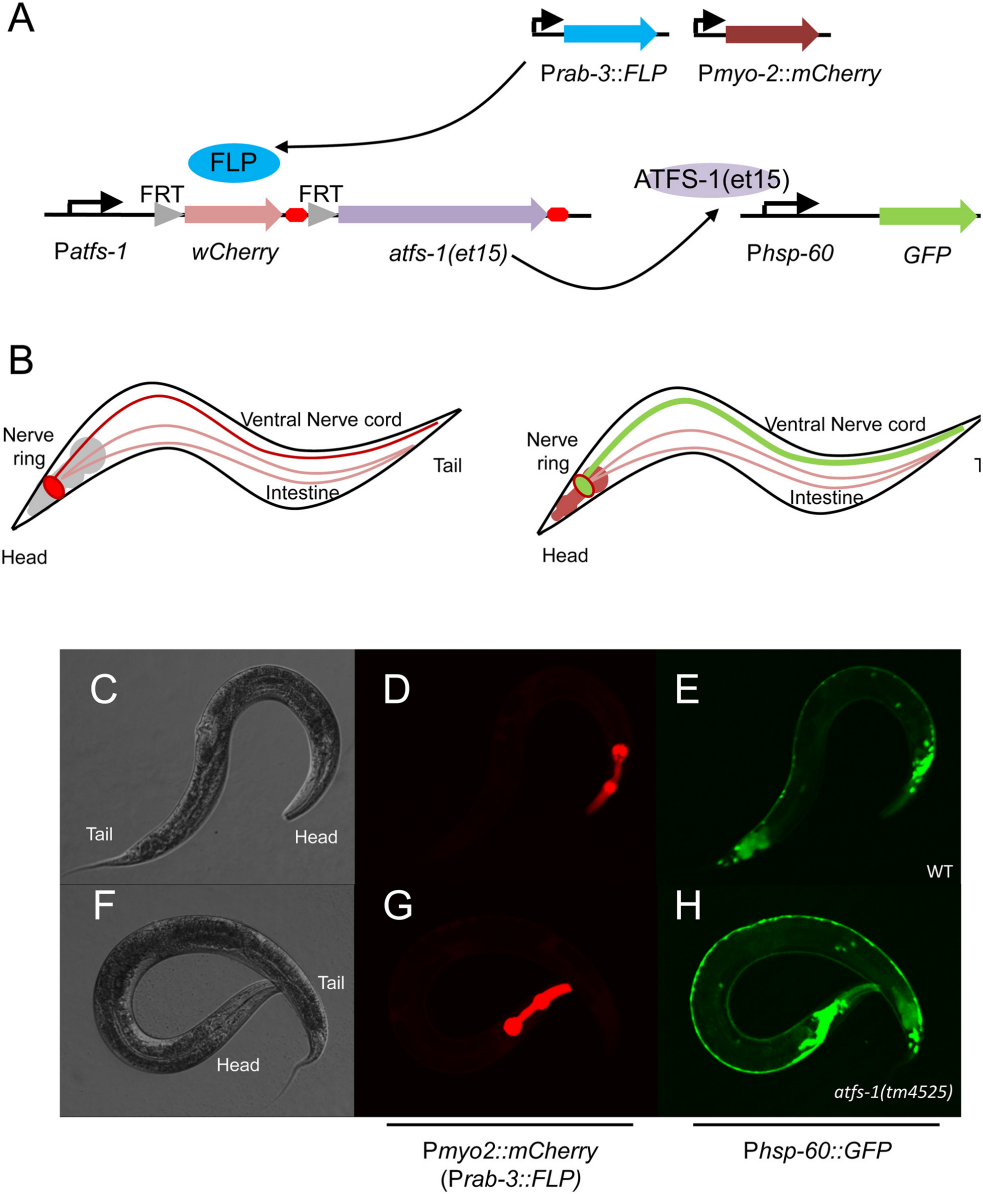
FLP-out *atfs-1(gf)* and trans-cellular UPR^mt^ signaling. A.) Schematic showing the relevant genetic constructs and the activity of the *flp* and *atfs-1* gene products on their target promoters. B.) Schematic representing UPR^mt^ activation in neurons (green) in the presence of P*rab-3*::*FLP* (red). The intestine is denoted in pink, as labeled, and activation of the UPR^mt^ is notably absent in this tissue. C-E.) Transmission and fluorescent photomicrographs of cell-autonomous UPR^mt^ activation in a genomic *atfs-1(+)* background and in a F-H.) *atfs-1(4525)* background. Note that distal tissues do not express the P*hsp-60*::*GFP* transgene, regardless of genetic background.

Next, the *atfs-1(et15)* gf missense mutation was incorporated into the MosSCI transgene, to generate a cell-specific compensatory response in the absence of stress. When FLP recombinase was expressed using the pan-neural promoter *rab-3* (Fig 5A), we observed robust activation of the UPR^mt^ *hsp-60*::*GFP* reporter gene in neurons (Fig 5C–5H). We predicted that remote activation of the UPR^mt^ would require *atfs-1* acting in distal tissues, and so we expressed the MosSCI/FLP transgene in a genomic *atfs-1(lf)* mutant background as a presumed negative control. However, we found that regardless of whether the MosSCI *atfs-1(gf)* transgene was in a genomic *atfs-1(lf)* or *atfs-1(+)* background, the UPR^mt^ was not activated in distal tissues (Fig 5E and 5H). The lack of remote UPR^mt^ activation in our model was not due to an inability of distal tissues to sense mitochondrial proteostasis, as they responded normally to EtBr (S2 and S3 Figs). Hence, restricted expression of ATFS-1(gf) can elicit a cell-autonomous UPR^mt^, but cannot trigger its remote activation in other cells.

**Fig 6:**
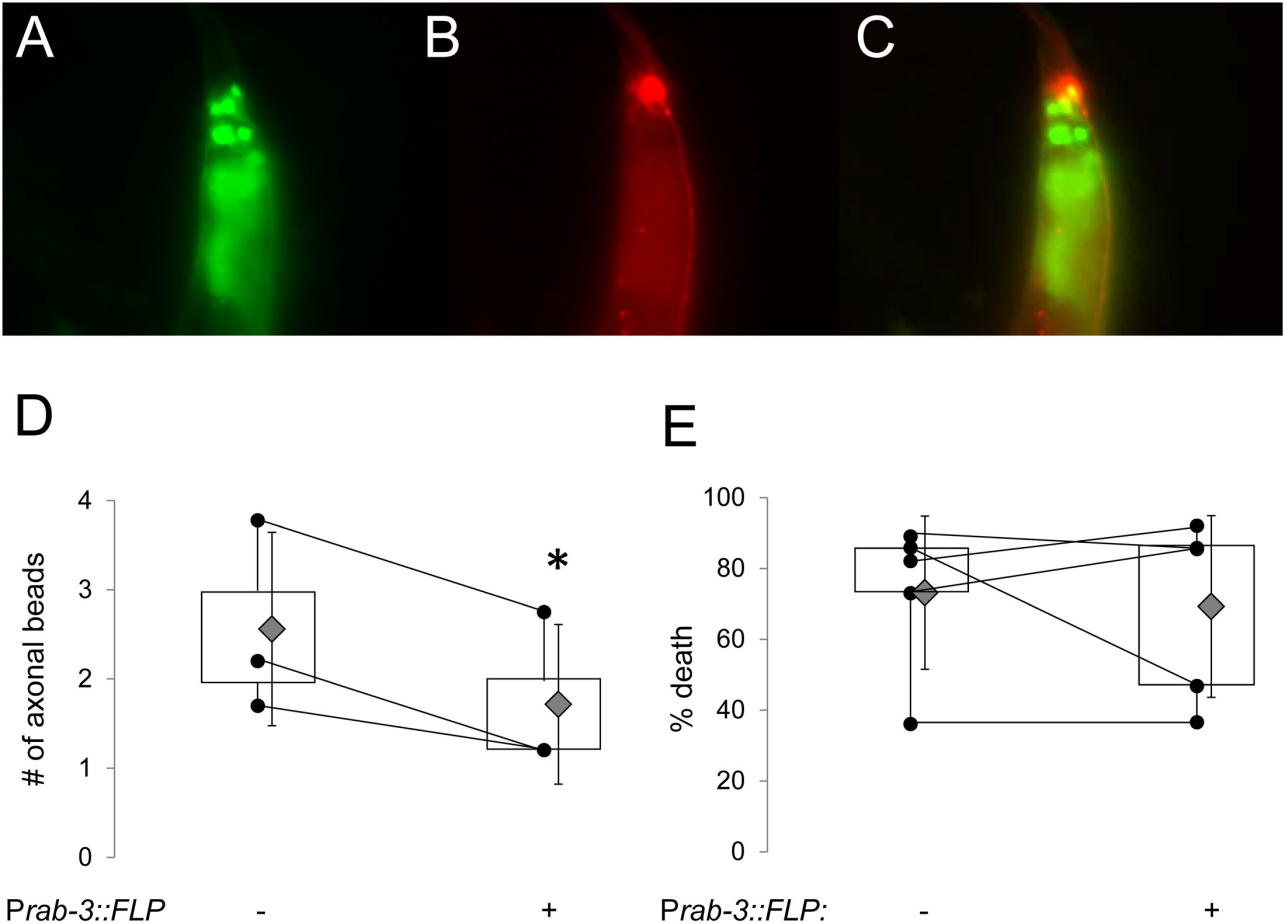
Protecting neurons from local anoxia-reperfusion injury in the *atfs-1(gf)* FLP out worm does not reduce A-R death. Representative fluorescent photomicrographs of a FLP out *atfs-1(et15)* transgenic worm with neuronal FLP (P*rab-3*::*FLP*) expressing A.) the UPR^mt^ reporter P*hsp60*::*GFP* (green) and B.) the neuronal reporter P*mec-4*::*mCherry* in mechanosensory neurons. Labeling of the PLM cell processes can be clearly distinguished over background P*atfs-1*::*wCherry* from the un-excised promoter interruption in other cells. C.) Merge indicating co-localization of UPR^mt^ activation and P*mec-4*::*mCherry*. D.) Expression of the FLP out *atfs-1(et15)* MosSCI transgene attenuates axonal damage following A-R (n = 3 independent trials of ~10 worms per genotype per trial), p*[Student’s t test] <0.05). The error is the standard deviation. E.) Box-and-whisker plot indicating that neuronal FLP out *atfs-1(et15)* MosSCI transgene does not reduce death following A-R (n = 5, p*[Student’s t test] <0.05). All data was obtained in an *atfs-1(+)* genomic background. Experimental replicates are shown as black dots; lines between replicates indicate that they were run on the same day. Grey diamonds are means with the error shown as standard deviations.

Finally, we asked whether the cell-autonomous UPR^mt^ activation observed in neurons reduced local A-R injury. The P*mec4*::mCherry reporter that was used to label mechanosensory neurons and their processes fluoresced much more brightly than the low level *wCherry* expressed from the single-copy promoter interruption (Fig 6B). We found that mechanosensory neurons from worms that exhibited FLP-mediated UPR^mt^ reporter activation (Fig 6A) were protected against axonal damage compared to their non-transgenic brood mates (*flp* null 2.6 ± 1.1; P*rab-3*:*flp* 1.7 ± 0.9, error is the SD, p = 0.04; Fig 6D). However, lethality following A-R was not influenced by the presence of the FLP transgene and did not correspond to UPR^mt^ reporter activation in neurons (*atfs-1(+)* background: *flp* null 73 ± 11%, P*rab-3*:*flp* 69 ± 13%, error is SEM, p = 0.69; Fig 6E). These results coincide with the lack of remote activation of the UPR^mt^ reporter (Fig 5E and 5H; S2 Fig), and hence, we hypothesize that neuronal mitochondrial stress itself is required to synchronize adaptive responses in other tissues.

## Discussion

It is clear from our results that pharmacologic or genetic pre-activation of the UPR^mt^ protects against 1.) A-R toxicity and 2.) axonal degeneration in neurons subject to sub-lethal A-R. We also find that mitochondrial dysfunction elicited through RNAi targeting of the ETC complex IV subunit *cco-1* in neurons can coordinate stress responses remotely to decrease A-R toxicity. Somewhat surprisingly however, neuronal expression of an *atfs-1(gf)* mutant did not reduce death, although it did function intrinsically to protect neurons themselves from axonal damage. Our results suggest that neuronal survival is not the major determinant of A-R survival and that *de facto* mitochondrial stress is required to coordinate remote stress responses through an unknown signal(s). However, our results do not discount a role for *atfs-1* acting in parallel to this unidentified signal, or being required in the target tissues independent of the signaling tissue. It’s interesting that the homeodomain-containing transcription factor DVE-1 and the small ubiquitin-like protein UBL-5 are both encoded by genes required for signaling the UPR^mt^ [3] and have been shown to work in parallel to *atfs-1*. In addition, mitochondrial function has been shown to regulate mTORC1 via ROS signaling (for review, see reference [45]), which together with its well established sensitivity to ATP may be central for metabolic adaptation and anoxic resistance and could potentially intertwine with UPR^mt^ signaling.

The UPR^mt^ has been suggested to be a surveillance response, whereby interruptions in core physiologic processes such as ETC function coordinate adaptation throughout the organism [12]. It seems intuitive that surveillance would best be served by a sentinel that’s selectively vulnerable to the type of damage that’s being detected (ie. a “first responder”). We speculate here that UPR^mt^ activation in the germline (or stem cells?) may act as a sentinel for mitochondrial damage. Ethidium bromide, which causes mitochondrial stress and induces the UPR^mt^, has been shown to preferentially affect germline maintenance in *C*. *elegans*. There are multiple precedents for germline-soma signaling. For example, translation (like mitochondrial function) is monitored as a surveillance mechanism [11], and lipid signals generated from the germline in response to translation inhibition can induce stress signaling in distal tissues [13]. The germline is well recognized to generate signals that lead to metabolic remodeling of somatic tissues [46–48]. Within this context, it’s quite interesting that stem cell maintenance also requires a UPR^mt^ mediated metabolic checkpoint [49]. Finally, we note that a previous report has shown that *glp-1* mutants which do not generate oocytes have altered fat stores and are greatly protected from A-R [50].

Genetic manipulations that impair mitochondrial function in *C*. *elegans* also alter lifespan [51]. Similarly, interventions that support protein folding are generally protective against low oxygen conditions and also extend lifespan [52]. One common denominator in these processes is that they rely on mitochondrial ROS in one capacity or another. However, an important difference is highlighted by recent work indicating that *atfs-1* is dispensable for *cco-1* RNAi mediated longevity [53]. In contrast, our work and that of others [27] indicate that protection from A-R injury via the UPR^mt^ requires *atfs-1* and, at least cell autonomously, that an ATFS-1(gf) that favors nuclear targeting is sufficient to reduce A-R death. Undoubtedly there are multiple signaling pathways that respond to mitochondrial stress, and the genetic requirements for protection from stress may overlap, but are likely to have insult specific components.

The UPR^mt^ like other compartment specific unfolded protein responses is aimed at restoring proteostasis in the face of stress. A general concept that emerges from multiple studies of stress is that adaptive signaling is generally beneficial in the short term, but chronic activation of these pathways is maladaptive. However, it’s still unclear whether this holds true for the UPR^mt^. For example, it’s been established that pre-activation of the UPR^mt^ protects against statin toxicity [42] and *Pseudomonas* infection [10]. In addition, several *C*. *elegans* mutants in electron transport chain (ETC) subunits require *atfs-1* for survival [16], suggesting that at least within the context of mitochondrial dysfunction any potential drawbacks of chronic activation are outweighed by the benefits. So, can we conclude that the UPR^mt^ is strictly beneficial?

Unfortunately, there are several observations suggesting that this is not as straightforward as would appear, and probably depends upon the degree of activation. Treatment with *spg-7(RNAi)* can cause larval maturation to stall, as can high concentrations of EtBr, both of which are means of activating the UPR^mt^. One might argue that these phenotypes result from excess mitochondrial dysfunction that cannot be compensated for by activation of the UPR^mt^. However, it is worth noting that the *atfs-1(et15)* gf mutant is generally smaller and matures more slowly than wildtype worms and displays reduced fecundity [42]. In addition, worms expressing an a nuclear targeted ATFS-1 with a deletion of the mitochondrial targeting sequence(Δ1-32.myc ATFS-1) similarly develop slower than worms expressing a wildtype ATFS-1 [16]. This may result from chronic nuclear activation, or alternatively from reduced ATFS-1 function in the mitochondria, whose genome has also been shown to be regulated by this transcription factor [15]. Hence, while there’s reason to believe that the UPR^mt^ may be primarily beneficial, further work is required to delineate the downside of chronic activation.

Collectively, our results demonstrate that *atfs-1* and the UPR^mt^ can influence A-R damage and survival. We further hypothesize that mitochondrial stress activates an as-of-yet unidentified signaling pathway(s) to coordinate adaptive responses throughout the organism. While the mammalian ATFS-1 ortholog has yet to be identified, the similarities between the UPR^mt^ in mammals and worms (for review, see reference [54] and the strong protection from A-R elicited by activation of the UPR^mt^ warrant further work on this topic.

## Supporting Information

S1 Fig UPR^mt^ activation by ethidium bromide reduces death following anoxia-reperfusion and requires *atfs-1*. A.) Box-and-whisker plot of A-R toxicity following treatment with 30 μg/ml EtBr to activate the UPR^mt^ (n = 8, p*[Student’s t test] <0.05) B.) *atfs-1(tm4525)* mutants fail to exhibit EtBr mediated protection (n = 5, p*[Student’s t test] <0.05). Grey diamonds are means with the error shown as standard deviations.
(PDF)

S2 Fig Distal tissues are capable of mounting an UPR^mt^ in the neuron specific *atfs-1(gf)* FLP-out transgenic model. Representative fluorescent photomicrographs of MosSCI FLP-out *atfs-1(gf)* transgenic worms grown on (A, C.) control plates and (B, D.) plates containing 30μg/mL EtBr. The transgenic marker for FLP (P*myo-2*::*mCherry*) is shown in panels A and B, while activation of the UPR^mt^ reporter P*hsp-60*::GFP is shown in panels C and D. The genomic background is *atfs-1(+)*. These data indicate that failure to activate distal tissue stress responses in this strain does not result from an intrinsic dysfunction.
(PDF)

S3 Fig Distal tissues remain responsive to UPR^mt^ inducers in the neuron specific FLP-out transgenic model. Representative photomicrographs of MosSCI FLP-out *atfs-1(gf)* transgenic worms grown on (A, C, F, H.) control plates and (B, D, E, G, I, J) plates containing 30μg/mL EtBr. Transmitted light images are shown in panels A-E, while fluorescent images are shown in panels F-J. Relevant information is labeled at the top of the figure. Note that the strain in panels E and J lacks the UPR^mt^ reporter P*hsp-60*::GFP and that the MosSCI FLP-out *atfs-1(gf)* is fused to GFP. These panels have been overexposed. The failure to detect either mitochondrial or nuclear GFP (the green is autofluorescence) exemplifies the low expression level of a single copy transgene and its likely degradation under basal conditions.
(PDF)

S1 Table Raw data. This is an Excel spreadsheet with the raw data organized by figure. Technical and experimental replicates are indicated.
(XLSX)

## Abbreviations

UPR^mt^: mitochondrial unfolded protein response
ETC: electron transport chain
I-R: ischemia reperfusion
MosSCI: Mos-mediated Single Copy gene Insertion
RNAi: RNA interference
NGM: normal growth media
gf: gain-of-function
lf: loss-of-function
EtBr: ethidium bromide
