# Supplementary figures and images for: The Mitochondrial Unfolded Protein Response Protects against Anoxia in Caenorhabditis elegans
Source: PLoS One. 2016 Jul 26;11(7):e0159989. doi: 10.1371/journal.pone.0159989 (PMC4961406; doi:10.1371/journal.pone.0159989)

S1 Fig

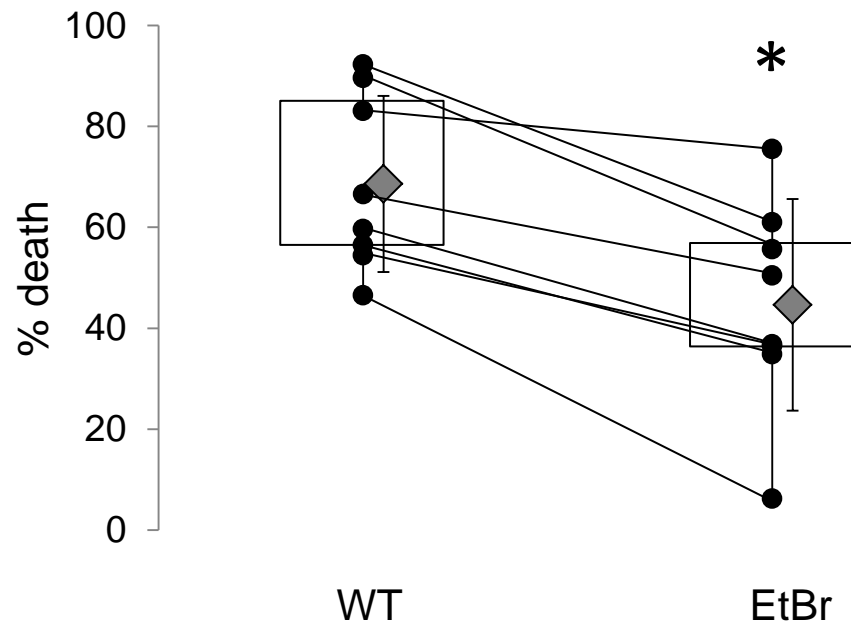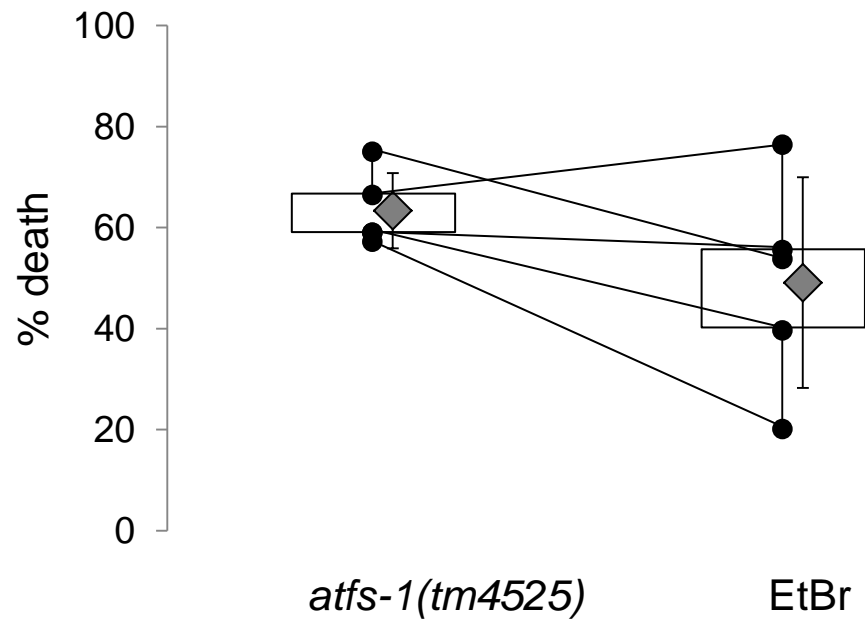

Supplement: S1 Fig — A.) Box-and-whisker plot of A-R toxicity following treatment with 30 μg/ml EtBr to activate the UPRmt (n = 8, p*[Student’s t test] <0.05) B.) atfs-1(tm4525) mutants fail to exhibit EtBr mediated protection (n = 5, p*[Student’s t test] <0.05). Grey diamonds are means with the error shown as standard deviations. (PDF) [file pone.0159989.s001.pdf]

S2 Fig.

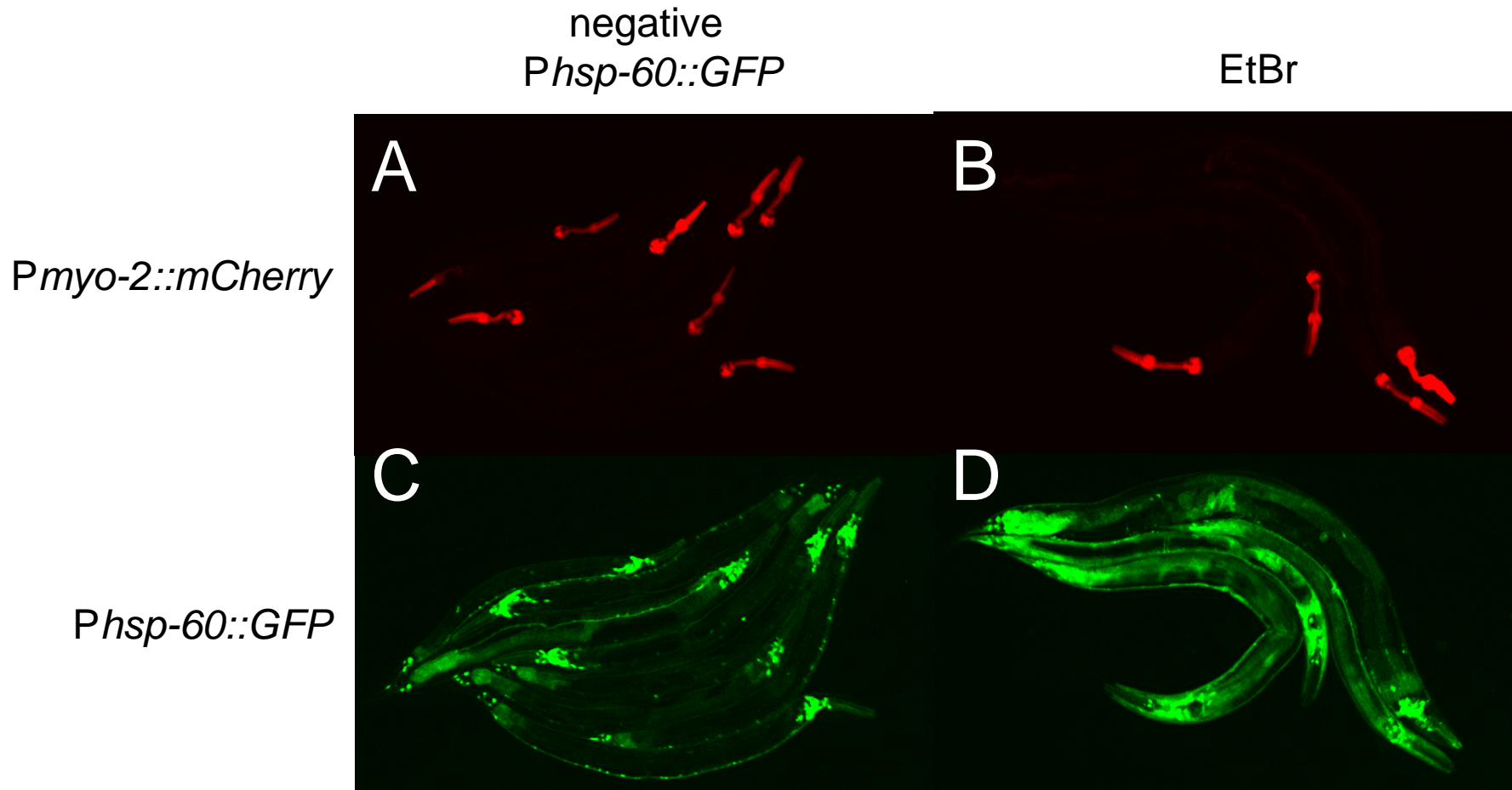

Supplement: S2 Fig — Representative fluorescent photomicrographs of MosSCI FLP-out atfs-1(gf) transgenic worms grown on (A, C.) control plates and (B, D.) plates containing 30μg/mL EtBr. The transgenic marker for FLP (Pmyo-2::mCherry) is shown in panels A and B, while activation of the UPRmt reporter Phsp-60::GFP is shown in panels C and D. The genomic background is atfs-1(+). These data indicate that failure to activate distal tissue stress responses in this strain does not result from an intrinsic dysfunction. (PDF) [file pone.0159989.s002.pdf]

S3 Fig.

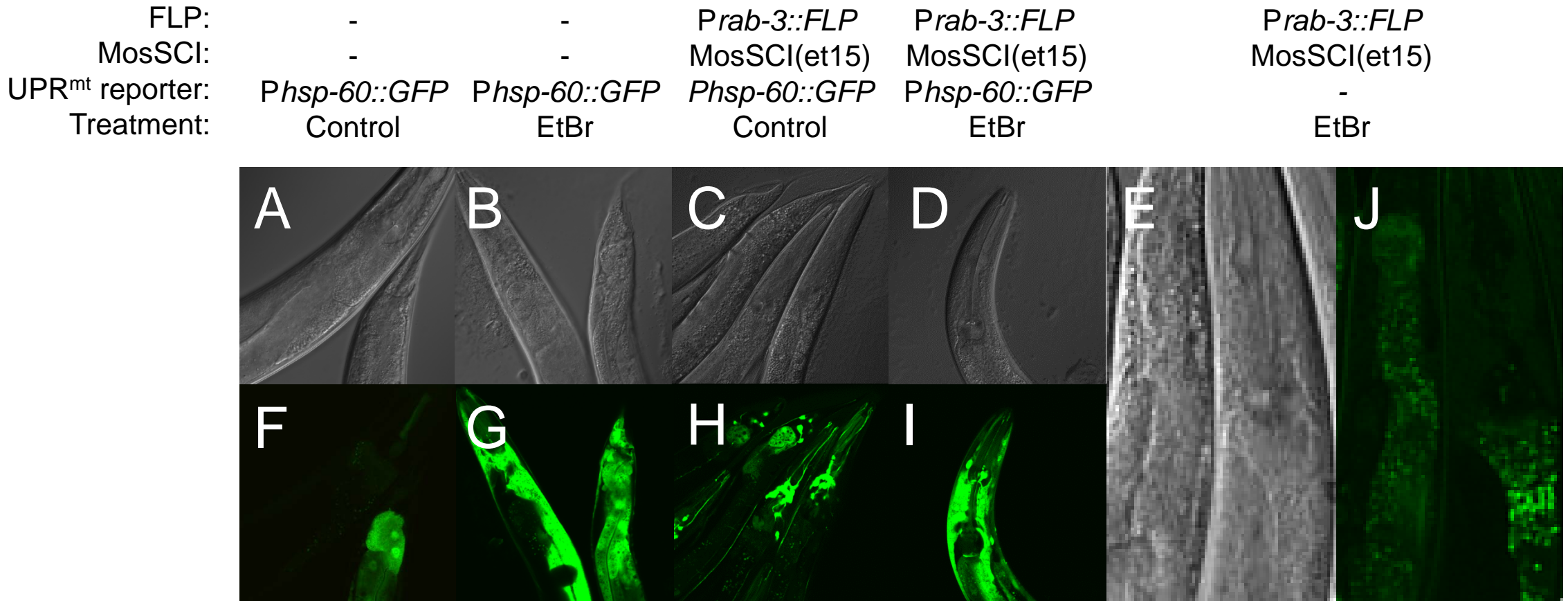

Supplement: S3 Fig — Representative photomicrographs of MosSCI FLP-out atfs-1(gf) transgenic worms grown on (A, C, F, H.) control plates and (B, D, E, G, I, J) plates containing 30μg/mL EtBr. Transmitted light images are shown in panels A-E, while fluorescent images are shown in panels F-J. Relevant information is labeled at the top of the figure. Note that the strain in panels E and J lacks the UPRmt reporter Phsp-60::GFP and that the MosSCI FLP-out atfs-1(gf) is fused to GFP. These panels have been overexposed. The failure to detect either mitochondrial or nuclear GFP (the green is autofluorescence) exemplifies the low expression level of a single copy transgene and its likely degradation under basal conditions. (PDF) [file pone.0159989.s003.pdf]
